# Controlling fetal stress for preventing adverse health conditions in neonates and children

**DOI:** 10.3389/fpain.2024.1265069

**Published:** 2024-04-04

**Authors:** Valeria Calcaterra, Gianvincenzo Zuccotti, Gloria Pelizzo

**Affiliations:** ^1^ Pediatric and Adolescent Unit, Department of Internal Medicine, University of Pavia, Pavia, Italy; ^2^ Pediatric Department, Buzzi Children’s Hospital, Milan, Italy; ^3^ Department of Biomedical and Clinical Science, University of Milan, Milan, Italy

**Keywords:** fetal stress, neonates, children, adverse health conditions, prevention

## Introduction

According to the World Health Organization, stress is a natural human response that helps us address challenges and threats in our lives (1). The effective stressor may arise from stimuli which include physical (e.g., tissue injury/inflammation), situational (e.g., loud noise, peculiar smells) or learned (memories of a traumatic experience) cues. When an individual encounters a potentially harmful stressor, interconnected neuroendocrine circuits are activated (2). The properties of the stress show significant overlap with pain, in both conceptual and biological processes, thus stress and pain may be highly comorbid (3).

We note that brain centres, such as the structures of the limbic forebrain (e.g., the classic Papez circuit) (4) receive input representative of the cues noted above and integrate these cues resulting in the stress state phenotype and stimulate peripheral networks like the sympathetic- adrenal-medullary (SAM) axis and renin-angiotensin system, as well as the hypothalamic- pituitary-adrenal (HPA) axis. This leads to the release of various hormones and neuropeptides that regulate cardiovascular, metabolic, and immune responses to overcome the challenge (2).

Both acute and chronic stress may have an impact on brain development and later health conditions; the relationship between stressors and disease is affected by the severity, number, and persistence of the stressors, as well as by the individual's biological vulnerability, psychosocial resources (5). If the stress remains unresolved and disrupts the homeostasis, it can result in altered neuroendocrine parameters and subsequent illness (2). In this opinion paper we want to underline the keypoints of fetal stress control as crucial point for preventing adverse health conditions in neonates and children. Sharing the opinions has the power to make an opportunity to debate on the topic and to reflect on what strategies could be for managing stress.

## Stress control

Stress is a defensive mechanism. Only stress that surpasses an individual's capacity to adapt and cope has a negative effect. Persistent stress has been associated with changes in specific brain areas, leading to modifications in neuronal network function and neuroendocrine/immune imbalances that can contribute to various illnesses (6).

Stressors lead to activation of two major constituents of the stress system: the HPA axis secreting glucocorticoids and the SAM axis secreting noradrenaline and norepinephrine (7).

The HPA axis is a complex system of neuroendocrine pathways and feedback loops that function to maintain physiological homeostasis (8). The response to stressful stimuli involves different networks depending on whether it is a physical or psychological stressor. Stressors leads to activation of two major constituents of the stress system: the HPA axis secreting glucocorticoids and the SAM axis secreting noradrenaline and norepinephrine (7). The SAM swiftly responds to acute stress by modulating cardiovascular, respiratory, renal, and endocrine functions, whereas the HPA system orchestrates a prolonged response to stress through the actions of glucocorticoids. These systems interact to maintain homeostasis and support adaptation to stress. The HPA axis plays a crucial role in mediating fetal programming. While these stress responses may offer short-term advantages and enhance survival, alterations in gene expression patterns resulting from these responses may lead to modified reactions in adulthood and lay the groundwork for disease development later in life (9).

Physical stressors mainly activate structures related to vital functions control located on brainstem and hypothalamus; psychological stressors are perceived in an anticipatory condition, which may heavily rely on limbic structures and can be modulated by the reward system. In response to both physical and psychological stressors, hippocampus activation is also involved (8).

The HPA axis is highly susceptible to programming during development and glucocorticoids act as the primary mediators of HPA programming.

The central nervous system undergoes formation and differentiation in the first month of gestation, with key processes such as neurogenesis, neuronal migration, differentiation, synaptogenesis, apoptosis, and myelination occurring by mid-gestation (5). The hypothalamic-hypophyseal portal system begins developing as early as 11 weeks of gestation, and by 12–13 weeks, corticotropin-releasing hormone activity is present, increasing with gestational age (10). Since the fetal stress system is immature, it also relies on maternal and placental inputs as an endocrine network (11).

Peripheral and central nociceptive neurons of the SM axis are specified early in development; key pathways that control their genesis mature into postnatal life (12). Premature babies possess the nociceptive circuitry necessary to detect pain; however, their sensory systems are not yet fully developed. An imbalance between excitatory and inhibitory processes results in heightened nociceptive signaling within the central nervous system. Specifically, the central nervous system of premature neonates is notably susceptible to excitotoxicity (13). Moreover, there is a noteworthy connection between pain and oxidative stress in preterm neonates, supporting an increased vulnerability to stress response in these infants (14).

As documented by Goksan et al. (15) through fMRI studies, numerous brain regions responsible for pain encoding in adults exhibit activity in full-term newborn infants during the initial 7 days of life. This substantiates the proposition that infants can undergo both the sensory and affective dimensions of pain, underscoring the critical need for proficient clinical pain management.

The utilization of pain assessment tools, including behavioral and physiological responses (facial actions, body movements, cry, heart rate, respiratory rate, blood pressure, and oxygen saturation), therefore becomes particularly significant to quantify pain in nonverbal patients already in the early stages of life (14).

## Prenatal stress and developmental programming

Recent research has demonstrated that prenatal stress, induced by maternal and fetal physical, psychological, emotional, environmental stressors plays a significant role in developmental programming of health and disease. Growth and development *in utero* involve a multifaceted and dynamic process, necessitating the interaction of maternal and fetal components to ensure survival and optimal growth throughout gestation. When adverse factors are encountered during pregnancy, it is reasonable to expect a “stress” signal to be transmitted to the developing fetus. This signal triggers alterations in the structure and function of fetal tissues, affects the activity of the fetal HPA axis, and prompts behaviors geared towards promoting survival after birth (9); this adaptive response may contribute to irreversible alterations in tissue structure or function, significantly heightening susceptibility to disease thereafter.

Maternal stressors, including maternal psychological distress, such as anxiety or depressive symptoms, domestic violence, bereavement, can impact the developing fetus by inducing physiological changes in the intrauterine environment. Maternal exposure to disrupted endocrine signaling (such as excessive glucocorticoid levels), or exposure to harmful chemical agents (such as cocaine, nicotine, or alcohol), along with complications during pregnancy and infections, can lead to adverse effects and unfavorable development of the unborn child (9). Additionally, as reported by Ucar et al. (16), maternal pre- and post-delivery stress levels might be different for vaginal or cesarean deliveries; vaginal delivery is associated with higher cortisol and higher heart rate variability, suggesting that cesarean section may compromise the activity of the HPA and autonomic nervous system axes.This altered activity of the stress axes in the mother has been correlated with the development of diseases in the offspring (16).

In contrast, conditions, including congenital malformations, acquired fetal infections, fetal surgery directly affect the fetus in physical manner. The prenatal surgery represents a multifactorial stressor event for mother and fetus; it can be carried out with varying degrees of invasiveness and surgery-induced stress response can be affected by preoperative, perioperative and postoperative factors (17). Noise exposure on pregnancy may be also considered a stressors that affects both the fetus and the mother herself (18). Indeed, exposure to noise can elevate biological stress responses, potentially heightening the risk of stress-related prenatal effects (19).

Maternal and fetal stressors can act as major stressors during the pre/perinatal period, primarily triggering HPA axis activation (11, 20).

Excess glucocorticoid exposure during critical periods of development in glucocorticoid-responsive organs like the brain, liver, or pancreas can result in permanent physiological alterations and impact long-term health outcomes (2). Both experimental and clinical studies showed that exposure to prenatal maternal stress acts on regulation of HPA system and the serotonin pain inhibitory system, increasing pain sensitivity in offspring (21).

These effects of excess glucocorticoid depend on species, sex, age, timing and duration of exposure (15). While evidence suggesting specific periods of heightened vulnerability to stress during pregnancy remains inconsistent, vulnerability appears to be greater following stress exposure during the 5th and/or 6th month of pregnancy (9).

In prenatal period, the stress response increased with gestational age. Pain perception development, fetal maturation, and “pain memory” are probably associated with this increase (8, 9). Transgenerational effects of stress and glucocorticoids on HPA function and behaviors has been also reported (8, 9). Thus, controlling fetal stress, pain or non–pain-related, is crucial to limit negative impact on long-term health in the subject and multiple generations.

## Connections between prenatal stress and adverse neonatal and childhood health

Prenatal stress has been associated with various birth complications, including preterm labour, preterm delivery, low birth weight, shortened gestational length, and a range of long-term effects such as attachment difficulties, stress hyper-responsiveness, cardio-metabolic and neurological disorders, asthma, allergies, difficult temperament, and affective disorders (2, 22, 23).

Fetal stress exposure can directly affect health, development, and long-term function.

It influences fetal HPA activity and alters gene expression patterns and organ structures, increasing the risk of adult-onset diseases (24). Exposure to prenatal maternal stress impacts on behavioral sensitivity to a painful injury and it has been also suggested as a risk factor for chronic pain (21).

Moreover, stressors indirectly impact the mother, predisposing her to perinatal depression, compromising the quality of postnatal care, and negatively affecting the family environment (24). These factors play a crucial role in the motor and sensory development, temperament, cognitive abilities, and behavioural and emotional responses of the developing child (20).

Eventhough the mechanisms of programming by pre- and perinatal glucocorticoid exposure remains not fully elucidated, recent experimental studies have reported epigenetic modifications in offspring with exposures to glucocorticoids in the parent and grand parent generation (21). In particular, DNA methylation modifications and small noncoding RNAs as vectors for inter- and trans-generational transmission of epigenetic effects has been proposed (25).

Fetal hypoxia and oxidative stress could represent crucial players in cell modifications responsible for changes in fetal programming of HPA axis (22). Indeed, fetal hypoxia represents an immediate danger to intrauterine life, but also to the future life. Fetal hypoxia exposure to short periods leads to a blood flow redistribution to protect the organs, such as brain and heart (22). However, prolonged periods of fetal hypoxia could cause DNA lesions leading to mutations as well as a disruption in the epigenetic state of the cell.

The role of dysbiosis in stressed mothers on fetal programming of HPA axis should be also considered (26). As reported in animal model, the stress-induced dysbiotic maternal vaginal microbiota contributes to the changes in hypothalamic gene expression of offspring following adulthood stress, leading to the HPA axis dysregulation (27).

## Prevention and future directions

Exposure to prenatal maternal stress impacts adult behavioral outcomes. Currently, there are limited precautions in place to prevent fetal distress. Treatment options for stress during pregnancy are limited and depends and depend on the specific stressor. Preventive strategies may be adopted in case of exposure to disrupted endocrine signaling or harmful chemical agents or environmental insults. For psycological distress, pregnant patients and their health care providers must constantly weigh the benefits and potential risks of any given medication for the patient and the fetus (28, 29) There are also several nonpharmacologic treatment options for depression and stress during pregnancy, including interpersonal therapy, cognitive behavioral therapy, bright light therapy, massage therapy, acupuncture, mindfulness practice (30).

In instances of maternal and fetal physical stressors, novel surgical instruments for minimal invasive approach and anesthetic protocols must ensure maternal and fetal cardiovascular stability, sustained placental blood flow, minimal depression of fetal organ functions, and consequently, limit stress (31). Optimizing the treatment of intrauterine fetal infections must also be considered as a management strategy (32).

Increased understanding of the interactions among early-life stress, sex, and pain may lead to the identification of novel therapeutic targets and epigenetic drugs for the treatment of chronic pain disorders (33).

Recognizing the potential of early chronic stress to cause serious diseases is pivotal for understanding and preventing long-term illnesses. By focusing on the cumulative effects of chronic stress and life events from prenatal stages, clinicians and researchers can develop targeted stress-reduction approaches to reduce the prevalence of adverse neonatal and childhood morbidities. The improvement of the knowledge of the pathogenic mechanisms of developmental programming will allow strategies to prevent and/or reverse these effects. Identifying and validating biomarkers for early detection of fetal stress to will also pave the way for new preventative measures and treatment strategies.

## References

[1] Stress. Available online at: https://www.who.int/news-room/questions-and-answers/item/stress, (accessed July 19, 2023).

[2] Mariotti A. The effects of chronic stress on health: new insights into the molecular mechanisms of brain-body communication. Future Sci OA. (2015) 1(3):FSO23. 10.4155/fso.15.21 28031896 PMC5137920

[3] Abdallah CG Geha P. Chronic pain and chronic stress: two sides of the same coin? Chronic Stress (Thousand Oaks). (2017) 1:2470547017704763. 10.1177/2470547017704763 28795169 PMC5546756

[4] Papez JW. A proposed mechanism of emotion. Arch Neurol Psychiatry. (1937) 38:725–43. 10.1001/archneurpsyc.1937.02260220069003

[5] Schneiderman N Ironson G Siegel SD. Stress and health: psychological, behavioral, and biological determinants. Annu Rev Clin Psychol. (2005) 1:607–28. 10.1146/annurev.clinpsy.1.102803.144141 17716101 PMC2568977

[6] Thliveris JA Currie RW. Observations on the hypothalamo-hypophyseal portal vasculature in the developing human fetus. Am J Anat. (1980) 157(4):441–4. 10.1002/aja.1001570411 7405878

[7] Godoy LD Rossignoli MT Delfino-Pereira P Garcia-Cairasco N de Lima Umeoka EH. A comprehensive overview on stress neurobiology: basic concepts and clinical implications. Front Behav Neurosci. (2018) 12:127. 10.3389/fnbeh.2018.00127 30034327 PMC6043787

[8] Sheng JA Bales NJ Myers SA Bautista AI Roueinfar M Hale TM The hypothalamic-pituitary-adrenal axis: development, programming actions of hormones, and maternal-fetal interactions. Front Behav Neurosci. (2021) 14:601939. 10.3389/fnbeh.2020.601939 33519393 PMC7838595

[9] Xiong F Zhang L. Role of the hypothalamic-pituitary-adrenal axis in developmental programming of health and disease. Front Neuroendocrinol. (2013) 34(1):27–46. 10.1016/j.yfrne.2012.11.002 23200813 PMC3594480

[10] Ackland JF Ratter SJ Bourne GL Rees LH. Corticotrophin-releasing factor-like immunoreactivity and bioactivity of human fetal and adult hypothalami. J Endocrinol. (1986) 108(2):171–80. 10.1677/joe.0.1080171 3485168

[11] Pelizzo G Bellieni CV Dell'Oste C Zambaiti E Costanzo F Albertini R Fetal surgery and maternal cortisol response to stress. The myelomeningocele sheep model. J Matern Fetal Neonatal Med. (2016) 29(4):633–7. 10.3109/14767058.2015.1015412 25708491

[12] Fitzgerald M. The development of nociceptive circuits. Nat Rev Neurosci. (2005) 6:507–20. 10.1038/nrn1701 15995722

[13] Vinall J Grunau RE. Impact of repeated procedural pain-related stress in infants born very preterm. Pediatr Res. (2014) 75(5):584–7. 10.1038/pr.2014.16 24500615 PMC3992189

[14] Slater L Asmerom Y Boskovic DS Bahjri K Plank MS Angeles KR Procedural pain and oxidative stress in premature neonates. J Pain. (2012) 13(6):590–7. 10.1016/j.jpain.2012.03.010 22543043 PMC3367033

[15] Goksan S Hartley C Emery F Cockrill N Poorun R Moultrie F fMRI reveals neural activity overlap between adult and infant pain. Elife. (2015) 4:e06356. 10.7554/eLife.06356 Erratum in: Elife. 2015;4. doi: 10.7554/eLife.08663. 25895592 PMC4402596

[16] Uçar C Bülbül M Yıldız S. Cesarean delivery is associated with suppressed activities of the stress axes. Stress. (2022) 25(1):67–73. 10.1080/10253890.2021.2015318 34931594

[17] Finnerty CC Mabvuure NT Ali A Kozar RA Herndon DN. The surgically induced stress response. JPEN J Parenter Enteral Nutr. (2013) 37(5 Suppl):21S–9S. 10.1177/0148607113496117 24009246 PMC3920901

[18] Dzhambov AM Dimitrova DD Dimitrakova ED. Noise exposure during pregnancy, birth outcomes and fetal development: meta-analyses using quality effects model. Folia Med (Plovdiv). (2014) 56(3):204–14. 10.2478/folmed-2014-0030 25434079

[19] Sivakumaran K Ritonja JA Waseem H AlShenaibar L Morgan E Ahmadi SA Impact of noise exposure on risk of developing stress-related obstetric health effects: a systematic review and meta-analysis. Noise Health. (2022) 24(114):137–44. 10.4103/nah.nah_22_22 36124522 PMC9743309

[20] Coussons-Read ME. Effects of prenatal stress on pregnancy and human development: mechanisms and pathways. Obstet Med. (2013) 6(2):52–7. 10.1177/1753495X12473751 27757157 PMC5052760

[21] Knaepen L Pawluski JL Patijn J van Kleef M Tibboel D Joosten EA. Perinatal maternal stress and serotonin signaling: effects on pain sensitivity in offspring. Dev Psychobiol. (2014) 56(5):885–96. 10.1002/dev.21184 24311362

[22] McGowan PO Matthews SG. Prenatal stress, glucocorticoids, and developmental programming of the stress response. Endocrinology. (2018) 159(1):69–82. 10.1210/en.2017-00896 29136116

[23] Yüzen D Graf I Tallarek AC Hollwitz B Wiessner C Schleussner E Increased late preterm birth risk and altered uterine blood flow upon exposure to heat stress. EBioMedicine. (2023) 93:104651. 10.1016/j.ebiom.2023.104651 37355458 PMC10363435

[24] Premji SS Pana GS Cuncannon A Ronksley PE Dosani A Hayden KA Prenatal allostatic load and preterm birth: a systematic review. Front Psychol. (2022) 13:1004073. 10.3389/fpsyg.2022.1004073 36267082 PMC9577361

[25] Buthmann J Huang D Casaccia P O'Neill S Nomura Y Liu J. Prenatal exposure to a climate-related disaster results in changes of the placental transcriptome and infant temperament. Front Genet. (2022) 13:887619. 10.3389/fgene.2022.887619 35571026 PMC9099074

[26] Silvestro S Diomede F Chiricosta L Zingale VD Marconi GD Pizzicannella J The role of hypoxia in improving the therapeutic potential of mesenchymal stromal cells. A comparative study from healthy lung and congenital pulmonary airway malformations in infants. Front Bioeng Biotechnol. (2022) 10:868486. 10.3389/fbioe.2022.868486 35774062 PMC9237219

[27] Yeramilli V Cheddadi R Shah J Brawner K Martin C. A review of the impact of maternal prenatal stress on offspring Microbiota and metabolites. Metabolites. (2023) 13(4):535. 10.3390/metabo13040535 37110193 PMC10142778

[28] Patel SR Wisner KL. Decision making for depression treatment during pregnancy and the postpartum period. Depress Anxiety. (2011) 28(7):589–95. 10.1002/da.20844 21681871 PMC3128653

[29] Brandon AR Freeman MP. When she says “no” to medication: psychotherapy for antepartum depression. Curr Psychiatry Rep. (2011) 13(6):459–66. 10.1007/s11920-011-0230-2 21877161

[30] Porter AC Hunter S Noonan K Hoffman MC. A mindfulness application for reducing prenatal stress. Journal of Midwifery & Women’s Health. (2022) 67(4):442–7. 10.1111/jmwh.13359 PMC9540335 35403807

[31] Soma-Pillay P Nelson-Piercy C Tolppanen H Mebazaa A. Physiological changes in pregnancy. Cardiovasc J Afr. (2016) 27(2):89–94. 10.5830/CVJA-2016-021 27213856 PMC4928162

[32] Dadwal V Bhatt RK. Intrauterine fetal infections: do-able approaches. J. Fetal Med. (2020) 7(1):5–8. 10.1007/s40556-020-00252-9

[33] Grégoire S Jang SH Szyf M Stone LS. Prenatal maternal stress is associated with increased sensitivity to neuropathic pain and sex-specific changes in supraspinal mRNA expression of epigenetic- and stress-related genes in adulthood. Behav Brain Res. (2020) 380:112396. 10.1016/j.bbr.2019.112396 31786273

